# Heterogeneity in the association between prediabetes categories and reduction on glomerular filtration rate in a 5-year follow-up

**DOI:** 10.1038/s41598-022-11392-5

**Published:** 2022-05-05

**Authors:** Marjan Manouchehri, Lucía Cea-Soriano, Josep Franch-Nadal, Antonio Ruiz, Albert Goday, Rosa Villanueva, Javier Diez-Espino, Manel Mata-Cases, Carolina Giraldez-García, Enrique Regidor, The PREDAPS Study Group, Jesús Torrecilla, Lourdes Carrillo, José Mancera, Teresa Mur, Rosario Serrano, F. Javier García-Soidán, Gabriel Cuatrecasas, Dimas Igual, Ana Moreno, J. Manuel Millaruelo, Francisco Carramiñana, Manuel Antonio Ruiz, Francisco Carlos Pérez, Yon Iriarte, Ángela Lorenzo, María González, Beatriz Álvarez, Lourdes Barutell, Ma Soledad Mayayo, Mercedes Del Castillo, Emma Navarro, Fernando Malo, Ainoha Cambra, Rosa Magallón, Riánsares López, M. Ángel Gutiérrez, Luisa Gutiérrez, Carmen Boente, J. Javier Mediavilla, Luis Prieto, Luis Mendo, Ma José Mansilla, Francisco Javier Ortega, Antonia Borras, L. Gabriel Sánchez, J. Carlos Obaya, Margarita Alonso, Francisco García, Ángela Trinidad Gutiérrez, Ana Ma Hernández, Dulce Suárez, J. Carlos Álvarez, Isabel Sáenz, F. Javier Martínez, Ana Casorrán, Jazmín Ripoll, Alejandro Salanova, Ma Teresa Marín, Félix Gutiérrez, Jaime Innenaraty, Ma del Mar Álvarez, Sara Artola, Ma Jesús Bedoya, Santiago Poveda, Fernando Álvarez, Ma Jesús Brito, Rosario Iglesias, Francisca Paniagua, Pedro Nogales, Ángel Gómez, Jóse Félix Rubio, Ma Carmen Durán, Julio Sagredo, Ma Teresa Gijón, Ma Angeles Rollán, Pedro P. Pérez, Javier Gamarra, Francisco Carbonell, Luis García-Giralda, J. Joaquín Antón, Manuel De la Flor, Rosario Martínez, José Luis Pardo, Raquel Plana, Ramón Macía, Mercè Villaró, Carmen Babace, Concepción Blanco, Ángeles Jurado, José Luis Martín, Jorge Navarro, Gloria Sanz, Rafael Colas, Blanca Cordero, Cristina De Castro, Mercedes Ibáñez, Alicia Monzón, Nuria Porta, María del Carmen Gómez, Rafael Llanes, J. José Rodríguez, Esteban Granero, Manuel Sánchez, Juan Martínez, Patxi Ezkurra, Luis Ávila, Carlos De la Sen, Antonio Rodríguez, Pilar Buil, Paula Gabriel, Pilar Roura, Eduard Tarragó, Xavier Mundet, Remei Bosch, J. Carles González, Ma Isabel Bobé, Irene Ruiz, Flora López, Ana Martínez, Marti Birules, Oriol Armengol, Rosa De MarMiguel, Laura Romera, Belén Benito, Neus Piulats, Beatriz Bilbeny, J. José Cabré, Xavier Cos, Ramón Pujol, Mateu Seguí, Carmen Losada, A. De MaríaSantiago, Pedro Muñoz

**Affiliations:** 1 Department of Public Health and Maternal and Child Health, Faculty of Medicine, Universidad Complutense de Madrid, Pza. Ramón y Cajal, s/n. Ciudad Universitaria, 28040 Madrid, Spain; 2 Instituto de Investigación Sanitaria del Hospital Clínico San Carlos (IdISSC), Madrid, Spain; 3 redGDPS Foundation, Madrid, Spain; 4 Barcelona City Research Support Unit, University Institute for Research in Primary Care Jordi Gol, Barcelona, Spain; 5 CIBER Diabetes Y Enfermedades Metabólicas Asociadas, Madrid, Spain; 6 Department of Medicine, Universidad de Barcelona, Barcelona, Spain; 7 Centro de Salud Universitario Pinto, Madrid, Spain; 8 Universidad Europea de Madrid, Madrid, Spain; 9 Servicio de Endocrinología, Hospital del Mar, IMIM, Barcelona, Spain; 10 Department of Medicine, Universitat Autònoma de Barcelona, Barcelona, Spain; 11 CIBER Fisiopatología de La Obesidad Y Nutrición, Madrid, Spain; 12 Tafalla Health Center, Navarra, Spain; 13 Instituto de Investigación Sanitaria de Navarra (IDiSNA), Pamplona, Spain; 14 Servicio de Medicina Preventiva, Hospital Universitario del Tajo, Aranjuez (Madrid), Spain; 15 CIBER Epidemiología Y Salud Pública (CIBERESP), Madrid, Spain; 16 Centro de Salud Bombarda-Monsalud, Zaragoza, Spain; 17 Centro de Salud La Victoria de Acentejo, Santa Cruz de Tenerife, Spain; 18 Centro de Salud Ciudad Jardín, Málaga, Spain; 19 Centro de Atención Primaria Terrassa Sud, Barcelona, Spain; 20 Centro de Salud Martin de Vargas, Madrid, Spain; 21 Centro de Salud Porriño, Pontevedra, Spain; 22 Centro de Atención Primaria de Sarrià, Barcelona, Spain; 23 Centro de Atención Primaria Manuel Encinas, Cáceres, Spain; 24 Centro de Salud San Roque de Badajoz, Badajoz, Spain; 25 Centro de Salud Torrero La Paz, Zaragoza, Spain; 26 Centro de Salud Agost, Alicante, Spain; 27 Centro de Salud Aizarnazabal-Getaria, Guipúzcua, Spain; 28 Centro de Salud Alcalá de Guadaira, Madrid, Spain; 29 Centro de Salud Alcantarilla Sangonera, Murcia, Spain; 30 Centro de Salud Andrés Mellado, Madrid, Spain; 31 Centro de Salud Añaza, Santa Cruz de Tenerife, Spain; 32 Centro de Salud Ares, Coruña, Spain; 33 Centro de Salud Arrabal, Zaragoza, Spain; 34 Centro de Salud Artilleros, Madrid, Spain; 35 Centro de Salud Ávila Sur Oeste, Ávila, Spain; 36 Centro de Salud Beraun, Guipúzcoa, Spain; 37 Centro de Salud Burgos Rural, Burgos, Spain; 38 Centro de Salud Cáceres-La Mejostilla, Cáceres, Spain; 39 Centro de Salud Cadreita, Navarra, Spain; 40 Centro de Salud Campos-Lampreana, Zamora, Spain; 41 Centro de Salud Canal Salat, Baleares, Spain; 42 Centro de Salud Carballeda, Zamora, Spain; 43 Centro de Salud Chopera, Madrid, Spain; 44 Centro de Salud De La Eria, Asturias, Spain; 45 Centro de Salud Don Benito Este, Badajoz, Spain; 46 Centro de Salud El Calero, Las Palmas, Spain; 47 Centro de Salud Eras de Renueva, León, Spain; 48 Centro de Salud Espronceda, Madrid, Spain; 49 Centro de Salud Federica Monseny, Madrid, Spain; 50 Centro de Salud Fuente de San Luis, Valencia, Spain; 51 Centro de Salud General Ricardos, Madrid, Spain; 52 Centro de Salud Hereza Leganes, Madrid, Spain; 53 Centro de Salud Hereza, Madrid, Spain; 54 Centro de Salud Jumilla, Murcia, Spain; 55 Centro de Salud La Calzada 2, Asturias, Spain; 56 Centro de Salud La Matanza, Baleares, Spain; 57 Centro de Salud Lain Entralgo, Madrid, Spain; 58 Centro de Salud Las Águilas, Madrid, Spain; 59 Centro de Salud Lasarte, Guipúzcoa, Spain; 60 Centro de Salud Lavadores Vigo, Pontevedra, Spain; 61 Centro de Salud Los Rosales, Madrid, Spain; 62 Centro de Salud Los Yébenes, Madrid, Spain; 63 Centro de Salud Mallen, Sevilla, Spain; 64 Centro de Salud Medina del Campo Rural, Valladolid, Spain; 65 Centro de Salud Mislata, Valencia, Spain; 66 Centro de Salud Murcia Centro, Murcia, Spain; 67 Centro de Salud Ntra. Sra. de Gracia, Sevilla, Spain; 68 Centro de Salud Oñati, Guipúzcoa, Spain; 69 Centro de Salud Orihuela I, Alicante, Spain; 70 Centro de Salud Ponteareas, Pontevedra, Spain; 71 Centro de Salud Roces Montevil, Asturias, Spain; 72 Equipo de Atención Primaria Raval Sud, Barcelona, Spain; 73 Centro de Salud Rodríguez Paterna, La Rioja, Spain; 74 Centro de Salud Sada, A Coruña, Spain; 75 Centro de Salud Salvador Caballero, Granada, Spain; 76 Centro de Salud Salvador Pau, Valencia, Spain; 77 Centro de Salud San José Centro, Zaragoza, Spain; 78 Centro de Salud Santoña, Cantabria, Spain; 79 Centro de Salud Sta. María de Benquerencia, Toledo, Spain; 80 Centro de Salud Vandel, Madrid, Spain; 81 Centro de Salud Vecindario, Las Palmas, Spain; 82 Centro de Salud Vélez-Málaga Norte, Málaga, Spain; 83 Centro de Salud Villanueva de La Cañada, Madrid, Spain; 84 Centro de Salud Villaviciosa de Odón, Madrid, Spain; 85 Centro de Salud Vista Alegre Murcia, Murcia, Spain; 86 Centro de Salud Yecla, Murcia, Spain; 87 Centro de Salud Zumaia, Guipúzcoa, Spain; 88 Consultorio Almachar, Málaga, Spain; 89 Consultorio San Gabriel, Alicante, Spain; 90 Equipo de Atención Primaria Anglès, Girona, Spain; 91 Equipo de Atención Primaria Azpilagaña, Navarra, Spain; 92 Equipo de Atención Primaria Badia del Vallès, Barcelona, Spain; 93 Equipo de Atención Primaria Bellvitge, Barcelona, Spain; 94 Equipo de Atención Primaria El Carmel, Barcelona, Spain; 95 Equipo de Atención Primaria Girona 2, Girona, Spain; 96 Equipo de Atención Primaria Girona 3, Girona, Spain; 97 Equipo de Atención Primaria La Torrassa, Barcelona, Spain; 98 Equipo de Atención Primaria Martorell, Barcelona, Spain; 99 Equipo de Atención Primaria Poblenou, Barcelona, Spain; 100 Equipo de Atención Primaria Pubillas Casas, Barcelona, Spain; 101 Equipo de Atención Primaria Raval Nord, Barcelona, Spain; 102 Equipo de Atención Primaria Reus-1, Tarragona, Spain; 103 Equipo de Atención Primaria Sant Martí de Provençals, Barcelona, Spain; 104 Equipo de Atención Primaria Tremp, Lleida, Spain; 105 Unidad Básica de Salud Es Castell, Baleares, Spain; 106 Unidad de Gestión Clínica Adoratrices, Huelva, Spain; 107 Unidad Docente de Atención Familiar Y Comunitaria, Guadalajara, Spain; 108 Unidad Docente de Medicina Familiar Y Comunitaria, Cantabria, Spain

**Keywords:** Endocrinology, Medical research, Risk factors

## Abstract

Prediabetes and not just diabetes can cause kidney damage. This study assess the association of prediabetes with development of impaired renal function (IRF). We used data from PREDAPS prospective study a cohort of 1072 subjects with prediabetes and another cohort of 772 subjects without prediabetes were follow-up from 2012 to 2017. Prediabetes was defined according to American Association of Diabetes criteria. IRF was defined as having a glomerular filtration rate < 60 mL/min/1.73 m^2^. Incidence rates of IRF in both cohorts and in different categories of prediabetes, based on impaired glycosylated hemoglobin (HbA1c) and/or fasting plasma glucose (FPG), were calculated. Hazard ratios (HR) for the association of the prediabetes with IRF, adjusting for potential confounders, were estimated by Cox regression models. Incidence rates of IRF per 100 person-years were 1.72 (95% confidence interval [CI]: 1.34–2.21) and 1.79 (95%CI: 1.45–2.20) for those without and with prediabetes, respectively .The HR of IRF in subjects with prediabetes with respect to subjects without prediabetes was 0.76 (95% CI: 0. 54–1.07). Corresponding HRs for type of prediabetes was 0.68 (95%CI: 0.40–1.15) for those with both altered parameters, 0.68 (95%CI: 00.40–1.15) for those with only impaired HbA1c and 1.12 (95%CI: 0.68–1.85) for those with only impaired FPG. The present study reflects an overall trend towards a slightly decreased risk of IRF onset associated to prediabetes except for individuals with only isolated impaired FPG. Further studies are warranted to fully assess the renal progression of each group.

## Introduction

Approximately 422 million people globally suffer from diabetes globally and 1.6 million deaths are directly attributed to this each year^1,2^. Type 2 diabetes (T2D) is the most common, resulting as a result of increased insulin resistance. Diabetes is among the leading causes of chronic kidney failure around the world^2^. Impaired renal function (IRF) in patients with diabetes impose a significant health burden^3^. Deterioration of the renal function in combination with diabetes can lead to poorer health prognosis^4^.

It has been reported that up to 40% of patients in early stage of T2D demonstrate some degree of microvascular complication^5^. In addition, a high proportion of patients with diabetes are found to have non-diabetic renal disease (NDRD), being nephroangiosclerosis (NAS) the most frequent cause^6,7^. Metabolic changes associated with diabetes lead to glomerular hypertrophy, glomerulosclerosis, and tubulointerstitial inflammation and fibrosis^5^. In addition, according to various studies, one-third of adults with newly diagnosed diabetes mellitus already have kidney damage, suggesting that IRF may occur in pre-diabetic state^8^. The effect of hyperglycemia on the occurrence of IRF may start before glucose levels reaches diabetic ranges. Other diabetes-related microvascular complications, such as retinopathy and neuropathy have been described in some previous studies, in subjects with prediabetes^9,10^. On the other hand, it is known the high risk of kidney disease in the presence of microalbubnuria and the prevalence of microalbuminuria is higher in subjects with prediabetes than in subjects without alterations in glucose metabolism^11,12^.

Several authors have emphasized the early detection of IRF as an important component of its strategies for prevention of noncommunicable diseases^9^. However, the long-term influences of prediabetes on kidney function remains unknown. In order to fill major gaps in the role of prediabetes on IRF onset, the aim of this study was to evaluate the association between prediabetes and three diagnostic categories of prediabetes and incidence rate of IRF using a prospective cohort of individuals with prediabetes followed up by primary care physicians from Spain.

## Methods

### Study design

The Cohort Study in Primary Health Care on the Evolution of Patients with Prediabetes (PREDAPS), is a prospective study conducted by 125 Primary Care physicians at their practices from different provinces of Spain. The details of the cohort have been described elsewhere^13,14^. The age range of patients was between 30 and 74 years. The study period started in 2012 and continued up to the fifth annual follow-up visit in 2017. All individuals with the following criteria at baseline were excluded: diagnosis of diabetes, terminal disease, pregnancy, surgery, hospital admissions in the previous 3 months at study entry or any hematologic disease which could alter glycated hemoglobin A1c (HbA1c) values. The estimate glomerular filtration rate (eGFR) at baseline was calculated according to the Chronic Kidney Disease Epidemiology Collaboration (CKD-EPI). (10) A total of 92 subjects did not have measures eGFR at baseline and an additional 66 patients had values below 60 mL/min/1.73 m^2^ min which is already considered as a reduction; therefore, they were excluded due to this pre-existing condition. Final population included a total of 1844 subjects.

Study population was subdivided into two mutually exclusive cohorts according to glycemic parameters following American Diabetes Association (ADA) criteria: cohort of subjects with prediabetes (n = 1072) and cohort of subjects without prediabetes (N = 772). To define prediabetes, individuals met the ADA criteria for prediabetes: Considering having a Fasting Plasma glucose levels of 100–125 mg/dL and/or HbA1c range levels from 5.7 to 6.4%. Individuals were subdivided into three mutually exclusive diagnostic categories based on impaired glycemic parameters, collected at baseline. First category, included all subjects with only impaired fasting plasma glucose (FPG range levels: 100–125 mg/dL [5.6–6.9 mmol/L]), second category included all subjects with isolated impaired HbA1c (HbA1c range levels: 5.7–6.4% [39–47 mmol/mol]) and third category included subjects with both impaired glycemic parameters (FPG range levels: 100–125 mg/dL [5.6–6.9 mmol/L]) and HbA1c range levels: 5.7–6.4% [39–47 mmol/mol]))^15^.

**Figure 1:**
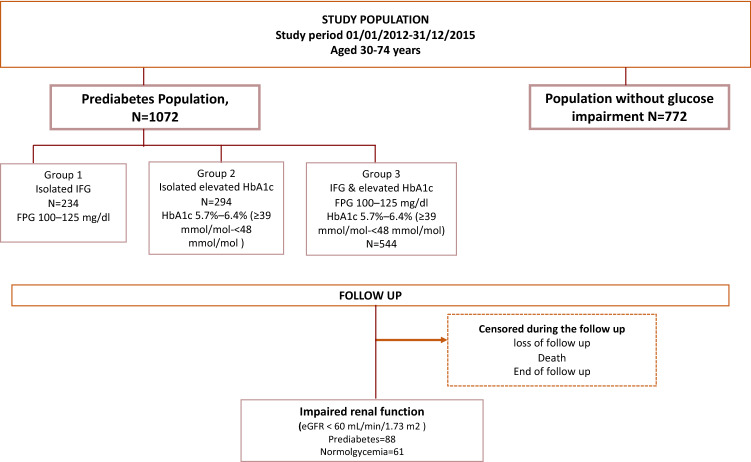
Flow chart of the study design.

Study period started on 2012 up to the fifth follow-up visit. Once meeting the eligibility criteria, individuals were followed up from baseline until the occurrence of one of the following end points: (i) IRF (ii) death, (iii) loss of follow up or (iv) end of study period (2017), whichever came first. In each subject, baseline serum creatinine was measured the day of enrolment in the study and additionally, each annual visit. IRF occurrence was measured at each annual visit and considered when a subject presented an eGFR < 60 mL/min/1.73 m2 during the follow up. IRF patients were immediately censored within the follow up (Fig. 1).

Subjects gave their written informed consent for participation. The study was classified by the Spanish Agency of Medicines and Medical Devices as a Non-Interventional (Observational) Post-Authorization Study, and the study protocol was approved by the Parc de Salut Mar Clinical Research Ethics Committee in Barcelona (2011-4274-I).

### Assessment of covariables

Data were collected at the first visit (baseline period). Information on biographical data, family history, comorbidities, demographical data (i.e. including social support and socio-economic position), lifestyle factors, and drug use were obtained from medical records of study subjects as well as a personal interview conducted by the physicians.

Comorbidities were categorized as follows. Hypertension (HTN) classified as systolic blood pressure ≥ 140 mmHg and/or diastolic blood pressure ≥ 90 mmHg, being treated by antihypertensive drugs or previous diagnosis of HTN. Hypercholesterolemia, as serum total cholesterol ≥ 250 mg/dL, HDL-C as < 40 mg/dL in men or < 50 mg/dL in women, and hypertriglyceridemia (HTG) as serum triglycerides ≥ 200 mg/dL.

Lifestyle factors were categorized as follows: Body mass index (BMI) between 25.0 and 29.9 kg/m^2^ classified as overweight, general obesity defined as BMI ≥ 30 kg/m^2^; abdominal obesity defined as waist circumference ≥ 102 cm in men and ≥ 88 cm in women. Smoking categories into smokers, ex-smokers and non-smokers; Alcohol consumption as: daily drinkers, occasional drinkers, and non-drinkers (never or former) which included ex-drinkers and teetotalers. Physical activity was classified according to World Health Organization (WHO) recommendations. Subjects followed the recommendations if they practiced more than 150 min per week of moderate aerobic physical activity, more than 75 min each week of vigorous aerobic physical activity or an equivalent combination^16^. For the adherence to Mediterranean diet (MedDiet), it was used as reference the definition followed in ATTICA study and their designed *Panagiotakos* score^17^. For each twenty types of studied nutrients, subjects responded the frequency of consumption: every day, more than three times a week, two times each week, once a week, less than once a week, never or rarely. Zero as a score in each meal was considered if the subject was having a less healthy diet and 4 was considered if the subject was having a very healthy diet. There were no missing values on demographic and clinical characteristics of participants at baseline.

### Statistical analysis

First of all, a descriptive analysis was conducted showing the distribution of the baseline characteristics among those who were classified in prediabetic cohort and those in the cohort without prediabetes. Continuous and count variables were described using mean (± standard deviation [SD]), median (quartiles) and 95% confidence intervals (95%CI). Incidence rate of IRF per 100 person-years together with 95%CI were calculated in each cohort. Incidence rate of IRF according to prediabetes categories was also calculated. Kaplan Meier survival functions with log rank test were performed to compare the survival distributions across each group. Cox proportional hazards analyses were used to estimate the hazard ratios (HR) with 95%CI for the association of covariables with incidence rate of IRF and for the association of prediabetes and diagnostic categories of prediabetes with reduction of incidence rate of IRF. Results of the association of prediabetes and diagnostic categories of prediabetes with incidence rate of IRF onset were shown for four levels of adjustment: model 1 (adjusted by age and sex), model 2 (model 1 plus adjusted by lifestyle variables such as smoking status, regular physical activity, high-risk alcohol consumption, adherence to MedDiet score, model 3 (model 2 plus adjusted by metabolic risk factors such as waist circumference, BMI, hypertension (HTN), total cholesterol, low HDL-cholesterol, triglycerides), and model 4 (model 3 plus adjusted by use of angiotensin converting enzyme inhibitors [ACEIs] or angiotensin II receptor blockers [ARBs]). Cox proportional hazards models assume that the HR is constant over time. It was verified graphically that this assumption was not violated since the observed and predicted value curves were similar. Likewise, the proportional-hazards assumption on the basis of Schoenfeld residuals test confirmed the findings obtained graphically. Interval censoring strategy was uses when information on time to event was not available due to loss to follow-up or non-occurrence of outcome event before the l end of the study. However, if the periodicity of examination is at a justified frequency, interval censored data were dealt with as point censor. Statistical analyses were performed using the STATA package version 12.0 (StataCorp LP, College Station, TX, USA).

### Ethics approval and consent to participate

The study was classified by the Spanish Drug and Health Product Agency as a Non-Interventional (Observational) Post-Authorization Study, and the protocol was approved by the Parc de Salut Mar Clinical Research Ethics Committee in Barcelona. Informed consent was obtained from all participants and/or their legal guardians.

### Method and Declaration Section

Authors confirm that all methods were performed in accordance with the relevant guidelines and regulations.

### Guarantor’s

Dr. Regidor is the guarantor of this work and, as such, had full access to all the data in the study and takes responsibility for the integrity of the data and the accuracy of the data analysis.

## Results

### Baseline characteristics

A total of 1,844 patients (95% Spanish origin) were included in the present study, from whom 1072 (58.1%) had prediabetes according to the ADA guidelines the mean age of prediabetic and normoglycemic groups were 59.1 (SD 9.3) and 56.6 (SD 10.3) years, respectively. At baseline, lifestyle factors were similarly distributed among patients with prediabetes compared with normoglycemia group. In terms of comorbidities, more than half of patients with prediabetes presented metabolic syndrome compared with 12.4% in the normoglycemic group. Likewise, the prevalence of HTN was higher in the prediabetic group. In particular, 36.9% of patients with prediabetes had treatment with ACEIs or ARBs drugs versus 23.6% in the normoglycemia group. For all metabolic parameters measured, prediabetic group presented a higher mean (*p* value < 0.01) with the exception of total cholesterol which the distribution was almost the same (210 mg/dL). Finally, the mean value of eGFR (mL/min/1.73 m^2^) was very similar across groups: 89.1 (SD: 13.7) for prediabetic cohort and 90.4 (SD:13.4) for normoglycemic cohort (Table 1).

**Table 1:** Demographic and clinical characteristics of participants at baseline.

Characteristics	Prediabetes	Normoglucose	*p* value
	(n = 1072)	(n = 772)	
Age (years), mean (SD)	59.1 (9.3)	56.6 (10.3)	< 0.001
Male, n (%)	536 (50.0)	358 (46.4)	0.068
**Smoking status, n(%)**			
Active smoker	182 (17.0)	170 (22.0)	0.003
Ex-smoker	409 (38.2)	244 (31.6)	
Never smoker	481 (44.9)	358 (46.4)	
Regular physical activity, n(%)	575 (53.7)	428 (55.4)	0.249
High-risk alcohol consumption, n(%)	140 (13.1)	83 (10.8)	0.078
Adherence Mediterranean diet score, n(%)	564 (52.6)	370 (47.9)	0.026
Daily consumption of fruit or vegetables, n(%)	919 (85.7)	653 (84.6)	0.269
Metabolic syndrome, n(%)	559 (52.1)	96 (12.4)	< 0.001
Waist circumference (cm), mean (SD)	100.1 (12.3)	93.0 (11.8)	< 0.001
BMI (kg/m^2^), mean (SD)	29.9 (4.8)	27.4 (4.4)	< 0.001
Fasting plasma glucose (mg/dL), mean (SD)	105.2 (10.9)	87.0 (7.2)	< 0.001
HbA1c (%), mean (SD)	5.8 (0.3)	5.3 (0.3)	< 0.001
**Hemoglobin (g/dL), n(%)**			
≥ 13.0	975 (91.0)	665 (86.1)	0.005
12.9–11.0	91 (8.5)	102 (13.2)	
≤ 10.9	6 (0.6)	5 (0.6)	
Hypertension, n(%)	708 (66.0)	359 (46.5)	< 0.001
SBP (mmHg), mean (SD)	134.6 (16.0)	128.3 (15.3)	< 0.001
DBP (mmHg), mean (SD)	81.2 (9.4)	79.0 (9.5)	< 0.001
Total colesterol (mg/dL), mean (SD)	209.9 (37.4)	211.0 (37.5)	0.532
HDL-cholesterol (mg/dL), mean (SD)	54.4 (14.3)	58.5 (15.6)	< 0.001
Non HDL-cholesterol (mg/dL), mean (SD)	129.6 (33.9)	129.8 (32.2)	0.899
Triglycerides (mg/dL), mean (SD)	132.3 (71.7)	114.4 (75.6)	< 0.001
Use of ACEIs or ARBs, n(%)	396 (36.9)	182 (23.6)	< 0.001
Creatinine(mg/dL), mean (SD)	0.8 (0.2)	0.8 (0.2)	0.760
eGFR (mL/min per 1.73 m^2^), mean (SD)	89.1 (13.7)	90.4 (13.4)	0.060

*SBP* Systolic blood pressure, *DBP* Diastolic blood pressure, *ACEIs* Angiotensin converting enzyme inhibitors, *ARBs* Angiotensin receptor blockers, *eGFR* Estimated glomerular filtration rate.

In addition, the urinary albumin could not be obtained in a large percentage of subjects (41%) and for this reason this variable was excluded from the analyses. However, the analysis of subjects with prediabetes (591) and subjects with normoglycemia (400) in whom this parameter was obtained, revealed no significant differences in the prevalence of microalbuminuria, whose magnitude was 7.5 and 5.8%, respectively (*p* = 0.297).

### Incidence rate of IRF overall and by prediabetes categories

**Figure 2:**
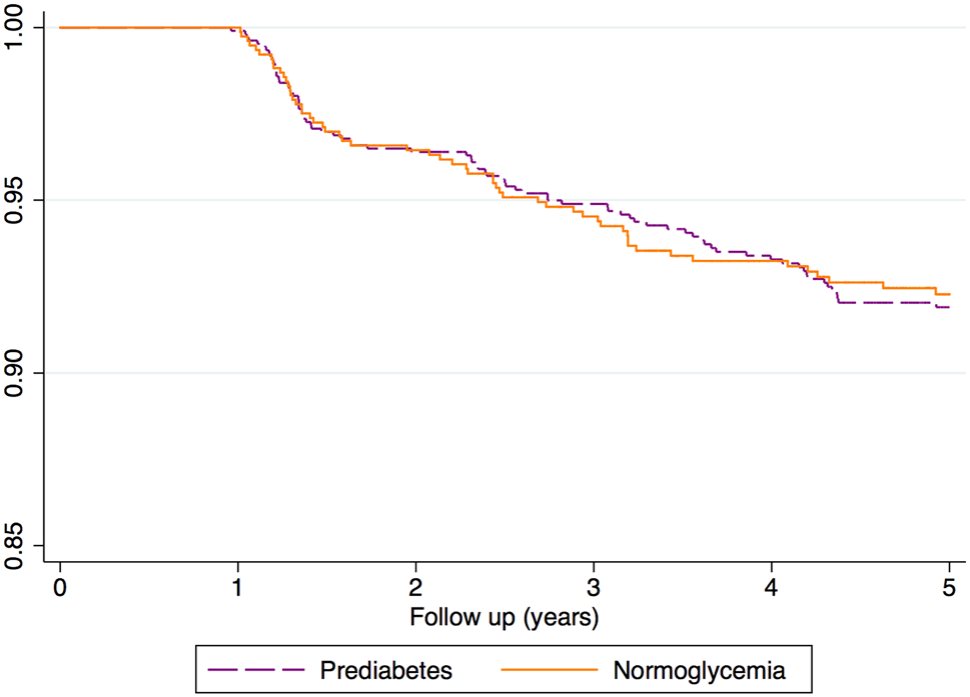
Kaplan–Meier survival estimate showing time to IRF onset according to prediabetes status.

**Figure 3:**
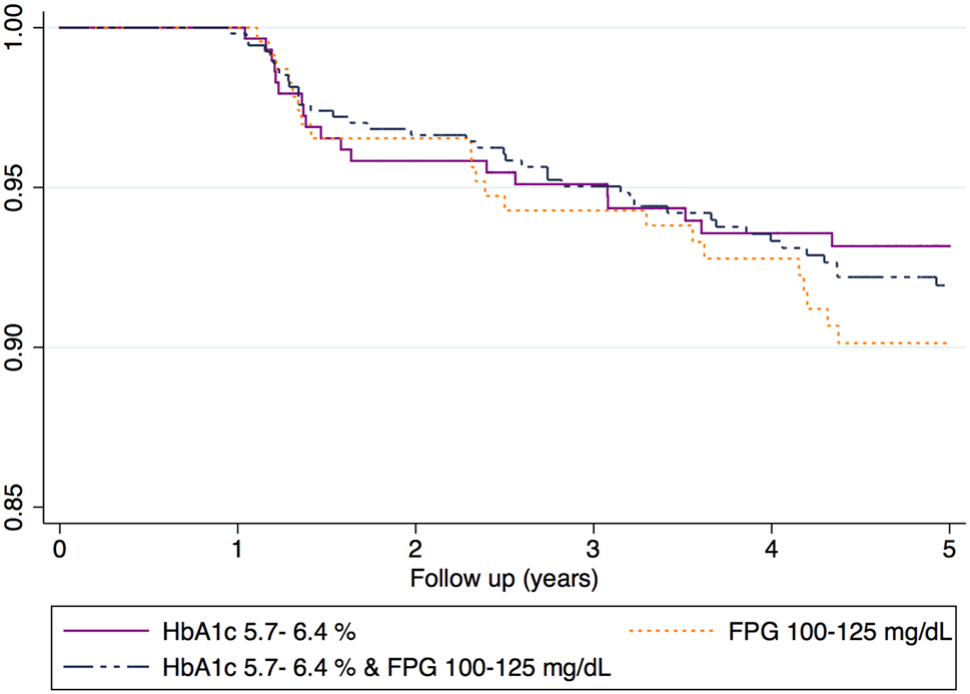
Kaplan–Meier survival estimate showing time to IRF onset according to type of prediabetes.

A total of 88 incident cases of IRF occurred in the prediabetic group and 61 cases occurred in the normoglycemic group. Incidence rates of IRF among the two study groups, overall and by prediabetes categories, are shown in Table 2. The overall incidence rate of IRF per 100 person-years was 1.72 (95%CI: 1.34–2.21) and 1.79 (95%CI: 1.45–2.20), log rank test *p* = 0.84. Focusing on the prediabetic group, the incidence of IRF was lower in the HbA1c 5.7–6.4% group (IR: 1.40 [95%CI: 0.89–2.19]) and highest among those with isolated FPG 100–125 mg/dL (IR: 2.06 [95%CI: 1.36–3.13]) log rank test *p* = 0.74. Figure 2 shows the Kaplan Meier survival function of IRF by type of cohort and Fig. 3 by prediabetes categories. To tests of proportional-hazards assumption, we estimated the Kaplan–Meier observed survival curves and compares them with the Cox predicted curves for the same variable. Supplemental Fig. 1 displays lines that the observed values and predicted values are close together.

**Table 2:** Five-year rate of incidence of impaired renal function per 100 person-years by cohort and prediabetes type.

	No. of cases	Person-years	Rate of incidence (100 person-years)
**COHORT**			
Prediabetes	88	4,928	1.79 (95%CI: 1.45–2.20)
Normoglycemia	61	3,555	1.72 (95%CI: 1.34–2.21)
**PREDIABETES TYPE**			
HbA1c 5.7–6.4%	19	1,359	1.40 (95%CI: 0.89–2.19)
FPG 100–125 mg/dL	22	1,066	2.06(95%CI: 1.36–3.13)
HbA1c 5.7–6.4% & FPG 100–125 mg/dL	47	2,503	1.88(95%CI: 1.41–2.50)

### Covariables and incidence rate of IRF

Table 3 shows the risk factors associated to IRF onset sex- and age-adjusted. There was a trend towards and increase risk of IRF according to age, for example those aged 50–64 years had a HR of 5.11 (95%CI: 1.85–14.12) and 16.48 (95% CI: 6.06–44.85) for those aged ≥ 65 years. Adherence to MedDiet showed a protective effect against reduction of eGFR (HR: 0.75 (95%CI: 0.54–1.04). On the contrary, metabolic conditions such as waist circumference ≥ 102 cm in men and ≥ 88 cm in women or having a BMI ≥ 30 were associated with an increased risk of reduction of eGFR (HR: 1.39 [95%CI: 0.54–1.04] and 1.21 [95%CI: 0.87–1.68], respectively). History of HTN showed a HR of 2.08 (95%CI: 1.38–3.12) and 1.82 (95% CI: 1.31–2.52) for use of ACEIs/ARBs but there was not association with metabolic syndrome (1.14 [95%CI: 0.82–1.59]). Having levels of HDL-cholesterol (mg/dL) as < 40 mg/dL in men or < 50 mg/dL was associated with a HR of IRF of 1.28(95%CI: 0.86–1.91).

**Table 3:** Association risk of comorbidities and lifestyle factors with risk of IRF development.

	HR	95% CI
**Age**		
30–49 years	1.00	
50–64 years	5.12	(1.85–14.15)
65–74 years	16.43	(6.04–44.73)
Sex (male)	1.09	(0.79–1.50)
**Smoking status**		
Active smoker	1.00	
Ex-smoker	0.93	(0.57–1.53)
Never smoker	0.78	(0.47–1.28)
Regular physical activity (no)	0.97	(0.70–1.35)
High-risk alcohol consumption (no)	1.28	(0.74–2.20)
Adherence Mediterranean diet score (no)	0.75	(0.54–1.04)
Daily consumption of fruit or vegetables (no)	1.18	(0.66–2.10)
Metabolic syndrome (no)	1.14	(0.82–1.59)
Waist circumference (cm)	1.39	(0.98–1.98)-
BMI ≥ 30 (kg/m^2^)	1.21	(0.87–1.68)
Hypertension, (no)	2.08	(1.38–3.12)
Total cholesterol ≥ 250 mg/dL*	1.15	(0.74–1.81)
Low HDL-cholesterol mg/dL*	1.28	(0.86–1.91)
Triglycerides ≥ 200 mg/dL	1.00	(0.68–1.45)
Use of ACEIs or ARBs (no)	1.82	(1.31–2.52)

Model 1 (sex- and age-adjusted).

*HDL-C of < 40 mg/dL in men or < 50 mg/dL in women.

### Association of prediabetes and diagnostic categories of prediabetes with incidence rate of IRF

Results are shown in Table 4. Using the cohort of subjects without prediabetes as reference, prediabetes was associated with a HR of IRF onset of 0.89 (95%CI: 0.64–1.24) when adjusting by age and sex. This estimate remained the same when adding lifestyle variables to the model and HR decreased to 0.76 (95%CI: 0.54–1.07) when adding metabolic conditions together with lifestyle factors. Of note, the estimate remained constant when adding on top of this model use of ACEIs or ARBs (Table 4). When evaluating the risk of IRF onset according to prediabetes diagnostic categories, a trend towards a decreased risk of IRF onset was observed in subjects with both parameters altered (FPG and HbA1c) and those with only impaired HbA1c levels, corresponding HR estimates were 0.68 (95%CI: 0.40–1.15) and 0.68 (95%CI: 0.40–1.15), respectively. However, subjects with only impaired FPG did not show any association (HR: 1.12 [95%CI: 0.68–1.85]).

**Table 4:** Hazard ratio of IRF associated to prediabetes and type of prediabetes using different models.

	Model 1		Model 2		Model 3		Model 4	
	HR	95%CI	HR	95%CI	HR	95%CI	HR	95%CI
Normoglycemia	Ref							
Prediabetes	0.89	(0.64–1.24)	0.90	(0.65–1.25)	0.76	(0. 54–1.07)	0.76	(0. 54–1.07)
HbA1c 5.7–6.4%	0.71	(0.43–1.20)	0.72	(0.43–1.21)	0.68	(0.40–1.14)	0.68	(0.40–1.15)
FPG 100–125 mg/dL	1.24	(0.76–2.03)	1.28	(0.78–2.11)	1.10	(0.66–1.82)	1.12	(0.68–1.85)
HbA1c: 5.7–6.4% & FPG 100–125 mg/dL	0.87	(0.59–1.27)	0.87	(0.59–1.28)	0.69	(0.46–1.03)	0.68	(0.45–1.02)

Model 1 (sex- and age-adjusted).

Model 2 (Model 1 plus adjusted by lifestyle variables (i.e. smoking status, regular physical activity, high-risk alcohol consumption, adherence Mediterranean diet (MedDiet) score, and daily consumption of fruit or vegetables).

Model 3 (Model 2 plus adjusted by metabolic risk factors (i.e. Waist circumference, BMI, hypertension, total cholesterol, HDL-cholesterol, triglycerides s).

Model 4 (Model 3 plus adjusted by use of ACEIs or ARBs).

## Discussion

Findings of this prospective cohort study reflect an overall trend towards a slightly decreased risk of IRF onset associated to prediabetes with an adjusted HR of 0.76. This finding is restricted to subjects who only had impaired HbA1c and those who had both parameters impaired: both groups represent 80% of the subjects with prediabetes and their adjusted HR was 0.76. Instead, subjects with only impaired FPG levels had a slightly increased risk (adjusted HR = 1.12).

A recent meta-analysis, including a total of eight cohort studies with subjects with impaired FPG as prediabetes criteria, has also reported a modest increased risk of IRF associated to impaired FPG^18^. It is known that hyperglycemia increases the production of reactive oxygen species, which lead to the accumulation of advanced glycation end products. This, in turn, activate intracellular signaling pathways such as protein kinase C and intensify the effects of the renin-angiotensin system^5^. This effect may lead to early onset of glomerular hyperfiltration and subsequently a decreased of IRF onset. In addition, eGFR has been reported to decrease faster in patients with hyperfiltration which might lead to kidney damage occurrence^19,20^. Although there is still controversy towards if hyperfiltration occurs in the early stages of hyperglycemia, several studies have found significant associations between hyperfiltration and prediabetes^21,22^. Specifically, it has been suggested how the prevalence of hyperfiltration increases with worsening stages of prediabetes^21^..

Several previous population-based studies of follow up have no found association of prediabetes with chronic kidney diseases nor with decreased GFR when using eGFR, after adjusting for risk factors^20,23,24^. Even in one of those studies prediabetes was associated with increased risk of hyperfiltration, but with reduced risk of having an mGFR < 60 ml/min/1.73 m2 at follow-up^24^. Our study presents as a novelty that the reduced risk of kidney damage can be observed only in some types of prediabetes. The reduced risk of IRF was concentrated among subjects who only had impaired HbA1c and those who had both parameters, while subjects with isolated impaired FPG showed a slightly increased risk of IRF, suggesting that these subjects might still preserve the renal function, and this is not via hyperfiltration. These results are consistent with a prior study using the same study population where it was found how individuals with impaired of both FPG and HbA1c had an OR of hyperfiltration of 1.69 (95% CI: 1.05–2.74) while there was no association among individuals with solely impaired FPG levels (25). Hyperfiltration was defined as an eGFR above the age and sex-specific 95th percentile^25^.

The early detection of IRF has been emphasized as an important component of its strategies for prevention of noncommunicable diseases^9^, as this has proven to improve outcomes for both individual and national healthcare economy. Since hyperfiltration is thought to be an early and proxy to reversible stage of kidney damage^26^ monitoring and identifying high risk prediabetic patients might result as an effective and cost-efficient preventive strategy. For example, both FPG and HbA1C levels can serve as chemical marker to identify early deterioration of IRF and avoid nephropathy. Another advantage of intensive blood glucose control in the prediabetic state can be seen in the long-term protective effect known as metabolic memory^27^. Thus, early intensive glycemic control could prevent irreversible damage that has been associated with hyperglycemia through closer monitoring of patients^26^. A 24% reduction in microvascular complications, including IRF, compared with tight glycemic control has been found in another study that followed up subjects with T2D for up to ten years^27^. Intensive glycemic control resulted in a 33% reduction in the risk of microproteinuria, proteinuria. Also a significant reduction in the proportion of patients with a doubling of the blood creatinine level (0.9% versus 3.5%) relative to the conventional therapy group was observed^27^.

This study reflects that it is possible to carry out a prospective study, with data obtained at the national level by primary care physicians during clinical practice. However, analytical determinations were made in different laboratories, which could have led to some misclassification. Given that each subject was assigned to the same laboratory during follow-up, this limitation should be non-differential in relation to the result, since an association between the methods used by specific laboratories and the development of IRF is unlikely.

This study used the IRF-EPI creatinine-based equation. This equation is more accurate and has less bias than the commonly used Diet Modification in Kidney Disease (MDRD) equation, especially at higher GFR levels^10^. In addition, GFR was estimated rather than using the gold standard of insulin clearance for this measure. Insulin clearance is more accurate than eGFR, but it is not cost-effective and it is an invasive method that is not used in clinical practice in Primary Care on a daily basis. However, analytical determinations were made in different laboratories, which could have led to some misclassification. Given that each subject was assigned to the same laboratory at baselines and during follow-up, this limitation should be non-differential in relation to the result, since an association between the methods used by specific laboratories and the development of IRF is unlikely. In addition, the urinary albumin could not be obtained in a large percentage of subjects (41%) and for this reason this variable was excluded from the analyses.

Finally, researchers were unable to determine a time-dependent variable. However, the vast majority of the factors considered in the present study are chronic conditions or long-term lifestyle factors not susceptible to a fast variation within the follow-up during the study period.

The current study did not show an increased risk of IRF onset associated to prediabetes, with the exception of those with isolated impaired FPG. Further studies are warranted to test the effect of these parameters can serve as chemical marker to identify early deterioration of IRF and avoid nephropathy. In any case, subjects with prediabetes could benefit from preventive measures that reduce their cardiovascular risk because cardiovascular and renal disease share common risk factors.

## Supplementary Information

Supplementary Information.

## Data Availability

The datasets used and/or analyzed during the current study available from the corresponding author on reasonable request.

## Abbreviations

ACEIs: Angiotensin converting enzyme inhibitors
ARBs: Angiotensin II receptor blockers
ADA: American diabetes association
BMI: Body mass index
IRF: Impaired renal function
Cr: Creatinine
DBP: Diastolic blood pressure
FPG: Fasting plasma glucose
GFR: Glomerular filtration rate
HbA1c: Glycated hemoglobin A1c
HDL: High-density lipoprotein
MedDiet: Mediterranean diet
NS: Not significant
OGTT: Oral glucose tolerance test
SD: Standard deviation
SBP: Systolic blood pressure
T2DM: Type 2 diabetes
WC: Waist circumference
