# Supplementary material for: Heterogeneity in the association between prediabetes categories and reduction on glomerular filtration rate in a 5-year follow-up
Source: Sci Rep. 2022 May 5;12:7373. doi: 10.1038/s41598-022-11392-5 (PMC9072306; doi:10.1038/s41598-022-11392-5)

**SUPPLEMENTAL FIGURES**

**Supplemental Figure 1**. Kaplan–Meier observed survival curves and Cox predicted curves for IRF among prediabetes population and population without glucose impairment


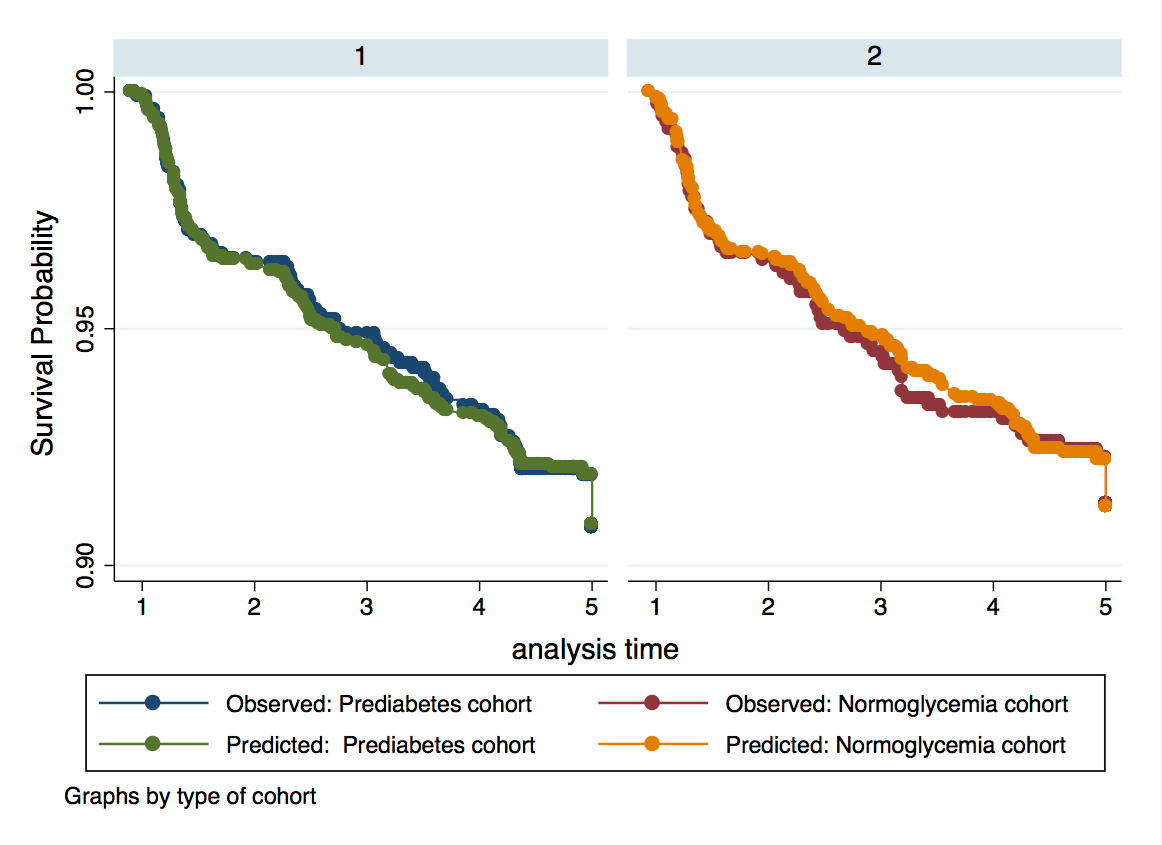

Supplement: Supplementary file 1 — Supplementary Information. [file 41598_2022_11392_MOESM1_ESM.docx]
